# Deregulation of XBP1 expression contributes to myocardial vascular endothelial growth factor‐A expression and angiogenesis during cardiac hypertrophy *in vivo*

**DOI:** 10.1111/acel.12460

**Published:** 2016-05-01

**Authors:** Quanlu Duan, Li Ni, Peihua Wang, Chen Chen, Lei Yang, Ben Ma, Wei Gong, Zhejun Cai, Ming‐Hui Zou, Dao Wen Wang

**Affiliations:** ^1^ Division of Cardiology, Department Internal Medicine, Tongji HospitalTongji Medical College, Huazhong University of Science and TechnologyWuhan430030People's Republic of China; ^2^ Center for Molecular and Translational MedicineGeorgia State UniversityAtlanta30303GAUSA

**Keywords:** angiogenesis, cardiac hypertrophy, unfolded protein response, vascular endothelial growth factor‐A, XBP1

## Abstract

Endoplasmic reticulum (ER) stress has been reported to be involved in many cardiovascular diseases such as atherosclerosis, diabetes, myocardial ischemia, and hypertension that ultimately result in heart failure. XBP1 is a key ER stress signal transducer and an important pro‐survival factor of the unfolded protein response (UPR) in mammalian cells. The aim of this study was to establish a role for XBP1 in the deregulation of pro‐angiogenic factor VEGF expression and potential regulatory mechanisms in hypertrophic and failing heart. Western blots showed that myocardial XBP1s protein was significantly increased in both isoproterenol (ISO)‐induced and pressure‐overload‐induced hypertrophic and failing heart compared to normal control. Furthermore, XBP1 silencing exacerbates ISO‐induced cardiac dysfunction along with a reduction of myocardial capillary density and cardiac expression of pro‐angiogenic factor VEGF‐A *in vivo*. Consistently, experiments in cultured cardiomyocytes H9c2 (2‐1) cells showed that UPR‐induced VEGF‐A upregulation was determined by XBP1 expression level. Importantly, VEGF‐A expression was increased in failing human heart tissue and blood samples and was correlated with the levels of XBP1. These results suggest that XBP1 regulates VEGF‐mediated cardiac angiogenesis, which contributes to the progression of adaptive hypertrophy, and might provide novel targets for prevention and treatment of heart failure.

**Abbreviations**
ATF: activating transcription factor
ER: endoplasmic reticulum
Grp: glucose‐regulated protein
IRE: inositol‐requiring kinase
ISO: isoproterenol
TAC: thoracic aorta constriction
TG: thapsigargin
TM: tunicamycin
UPR: unfolded protein response
VEGF‐A: vascular endothelial growth factor‐A
XBP: X‐box binding protein

## Introduction

Heart failure is the most common reason for hospitalization in the aging population and is a leading cause of mortality worldwide (Shah & Mann, 2011). Heart failure is the final common pathway of various cardiovascular diseases, including hypertension, myocardial ischemia or infarction, valvular heart disease, and inherited or acquired cardiomyopathies (Heineke & Molkentin, 2006). Cardiac hypertrophy occurs to enhance cardiac function as an adaptive response to increased workload, but prolonged cardiac hypertrophy causes heart failure (Heineke & Molkentin, 2006). During the development of cardiac hypertrophy, it has been postulated that cardiac angiogenesis, which is induced in the early adaptive phase, may be sufficient to reduce the disparity between the number of capillaries and the size of cardiomyocytes that develop to maintain cardiac function (Tomanek, 1990). In the maladaptive phase, the expression of angiogenic factors such as vascular endothelial growth factor‐A (VEGF‐A) is reduced (Giordano *et al*., 2001; Sano *et al*., 2007). In parallel, the number of CD31‐positive microvessels is reduced from compensatory hypertrophy with intact systolic function to symptomatic heart failure with depressed systolic function in patients with isolated valvular aortic stenosis and differing degrees of left ventricular systolic dysfunction (Hein *et al*., 2003). Thus, insufficient angiogenesis is considered as the key step for the transition of hypertrophic heart to heart failure (Shiojima *et al*., 2005). However, it remains poorly characterized that in hypertrophic hearts, the factors instigate angiogenesis and how angiogenesis becomes insufficient to maintain cardiac function in failing hearts.

The endoplasmic reticulum (ER) is a crucial organelle with essential roles in multiple cellular processes including intracellular calcium homeostasis, protein secretion, and lipid biosynthesis. When subjected to multiple stimulus and inadequate extracellular conditions including hypoxia, oxidative injury, cytotoxic drugs, etc., the resultant accumulation of unfolded proteins triggers an evolutionarily conserved series of signal transduction events termed the unfolded protein response (UPR) (Groenendyk *et al*., 2010). The UPR initiates three molecular compensatory mechanisms to promote cell survival and restore homeostasis to the ER (Okada *et al*., 2004). One of the major signaling pathways in the UPR is the IRE1α‐XBP1 pathway. Phosphorylation of IRE1α activates the endoribonuclease activity to splice XBP1 mRNA, induced by ATF6α, generating spliced XBP1 (XBP1s) mRNA encoding a potent transcription factor (Yoshida *et al*., 2001). XBP1s, is a key signal transducer in the ER stress response, also is a member of the CREB/ATF basic region‐leucine zipper family of transcription factors, which regulate genes involved in protein folding, glycosylation, ER‐associated degradation (ERAD), autophagy, lipid biogenesis, insulin secretion (Koong *et al*., 2006; Glimcher, 2010). XBP 1 is essential for survival under hypoxic conditions and is required for tumor growth (Romero‐Ramirez *et al*., 2004; Thuerauf *et al*., 2006). Specifically, accumulating evidence suggests that XBP1s exerts potential regulator effects in several cardiovascular diseases, including such atherosclerosis (Zeng *et al*., 2009), diabetes (Park *et al*., 2010; Lee *et al*., 2011; Zhou *et al*., 2011), myocardial ischemia (Wang *et al*., 2014) that ultimately results in heart failure. But so far, the expression trend and pathophysiological functions of XBP1 in cardiac hypertrophy and heart failure are still unknown. Recently, XBP1, as a key UPR‐inducible transcription factor (Glimcher, 2010), binds to the VEGF‐A promoter in response to ER stress in cancer cells (Roybal *et al*., 2005; Ghosh *et al*., 2010; Pereira *et al*., 2010). Furthermore, transient activation of XBP1 splicing may increase endothelial cell (EC) proliferation (Zeng *et al*., 2009), and endothelial cell‐specific knockout of XBP1 impaired the angiogenesis triggered by ischemia in mice (Zeng *et al*., 2013). Nevertheless, whether or not UPR such as XBP1 contributes to cardiac angiogenesis in hypertrophic hearts and heart failure remains to be determined.

Here, we hypothesized that ER stress transducer XBP1 may contribute to cardiac VEGF expression and then promote angiogenesis during the progression of cardiac hypertrophy. To test this hypothesis, we examined cardiac XBP1 expression and found that XBP1 is activated in animal hypertrophic heart model. Furthermore, our results have indicated that XBP1 activation is required for VEGF expression *in vitro* and consequent angiogenesis in the early stages of hypertrophic hearts *in vivo*, resulting in the progression of cardiac hypertrophy.

## Results

### Increased expression of XBP1s and ER stress in isoproterenol‐induced hypertrophic and failing heart

**Figure 1:**
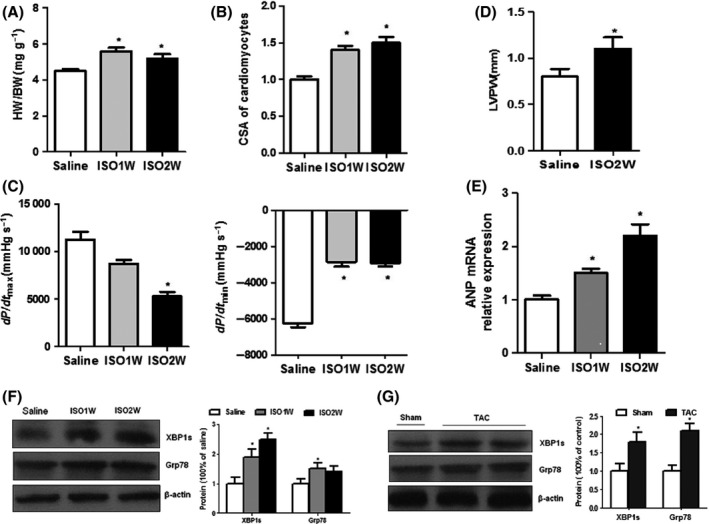
Cardiac XBP1 expression is increased in ISO infusion‐induced hypertrophic and failing heart. (A) Heart weight/body weight (HW/BW, grams) after ISO infusion. (B) Cross‐sectional area (CSA) of cardiomyocytes after ISO infusion. (C) Hemodynamic analysis of mice at 1 and 2 weeks of ISO infusion: (left) dP/dtmax (mmHg/s) and (right) dP/dtmin (mmHg/s). (D) Echocardiographic analysis of mice at 2 weeks of ISO infusion (LVPW, LV posterior wall thickness). (E) ANP mRNA expression in ISO‐treated mice. (F) Western blots of Grp78 and XBP1s in mouse hearts at 1 and 2 weeks of ISO infusion. (G) Western blot of Grp78 and XBP1s in mouse hearts after TAC treatment. Error bars indicate SEM. *n* = 6 for A–E. The blot is representative of at least four blots from four independent experiments; *P < 0.05 compared with control.

The mice hypertrophy model was established using isoproterenol (ISO) infusion. We first characterized this model using morphological, hemodynamic, and echocardiographic analysis. As depicted in Fig. 1A–B, heart weight/body weight (HW/BW) and cross‐sectional area (CSA) of cardiomyocytes significantly increased after ISO infusion. Consistently, hemodynamic analysis confirmed a reduction of dP/dtmax (mmHg/s) and dP/dtmin (mmHg/s) as early as 1 week after ISO infusion (Fig. 1C). Further, echocardiographic analysis showed that LV posterior wall thickness (LVPW) was increased after ISO infusion (Fig. 1D). Consistently, myocardial atrial natriuretic peptide (ANP) mRNA, a marker associated with cardiac hypertrophy and heart failure, was also significantly increased in mouse hearts after ISO infusion (Fig. 1E). Taken together, these results indicate that cardiac hypertrophy gradually developed from 1 to 2 weeks after ISO infusion and cardiac function declined thereafter. Then, we further determined whether ISO infusion caused ER stress in hypertrophic hearts *in vivo*. As depicted in Fig. 1F, protein levels of the ER chaperone GRP78 and UPR transcription factor XBP1 were increased as early as 1 week and continue to rise at 2 weeks after ISO infusion (Fig. 1F), suggesting that ISO infusion caused aberrant ER stress in the early phase of cardiac hypertrophy.

Thoracic aorta constriction (TAC) is another well established model to study hypertrophic and failing hearts. We next assayed the levels of ER stress, XBP1s in TAC‐induced hypertrophy in mice. As shown in Fig. 1G, cardiac expression of Grp78 and XBP1s were increased in the mice model at 2 week after TAC treatment (Fig. 1G). The levels of XBP1s in hearts were increased, as seen in ISO‐infused hypertrophic hearts. Taken together, our results suggest that XBP1 may play an important role in cardiac hypertrophy and heart failure.

### AAV9‐mediated XBP1 gene silencing exacerbates ISO‐induced cardiac dysfunction

**Figure 2:**
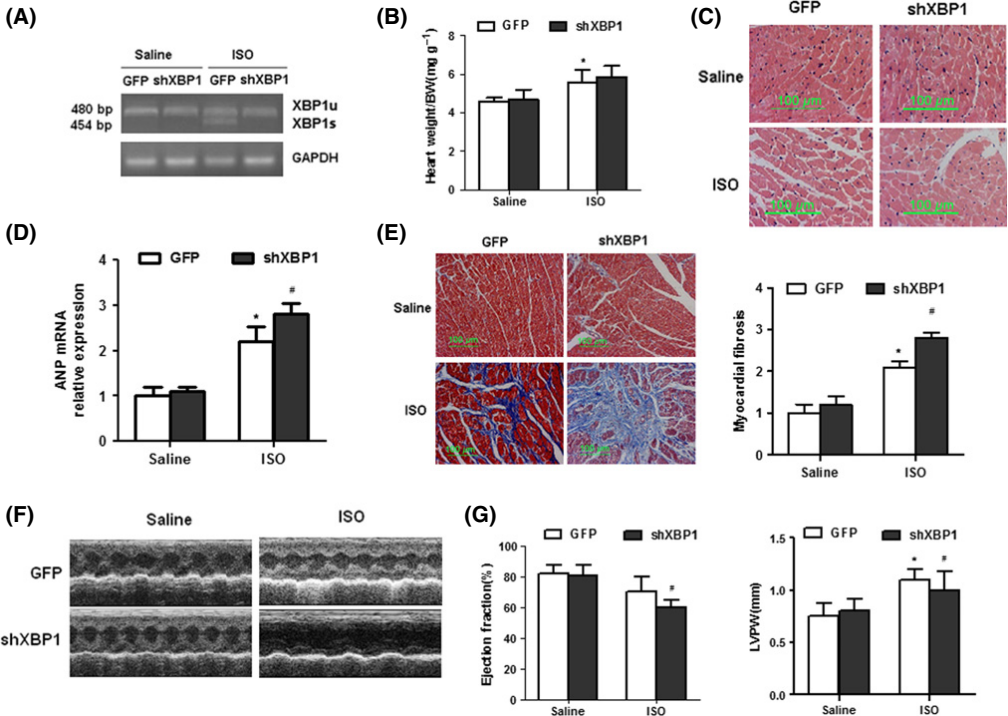
AAV9‐mediated‐inhibition expression of XBP1 exacerbates cardiac hypertrophy induced by ISO infusion. (A) Semiquantitative RT–PCR analysis of cardiac XBP1 mRNA expression in ISO‐infused mouse hearts after AAV‐shRNA XBP1 treatment. (B) Heart weight/body weight (HW/BW) in ISO‐treated mice after AAV‐XBP1‐shRNA treatment. (C) HE staining in ISO‐treated hearts after AAV‐shRNA XBP1 injection. (D) ANP mRNA expression in ISO‐treated mice after AAV‐XBP1‐shRNA treatment. (E) Representative sections for interstitial fibrosis (Meson‐stained). (F and G) Echocardiographic analysis of ISO‐treated mice after AAV‐shRNA XBP1 injection (LVPW, LV posterior wall thickness). Error bars indicate SEM. *P < 0.05 compared with GFP or #*P* < 0.05 compared with ISO+GFP. *n* = 8 for A–E.

Because XBP1 was markedly induced in hypertrophic and failing heart, we investigated the significance of XBP1 induction and its pathophysiological role in the development of heart failure. To address this issue, we silenced XBP1 by tail vein injection of the myocardium‐tropic adeno‐associated virus serotype 9 (AAV9) vector‐carrying XBP1 shRNA. Two weeks after tail intravenous injection of AAV9, the mice were subjected to ISO infusion to evaluate cardiac hypertrophy and cardiac functions. As expected, injection of XBP1 siRNA virus inhibited XBP1 expression *in vivo* (Fig. 2A). However, silencing of XBP1 did not alter basal cardiac size and function (Fig. 2B–G).

It was important to determine if XBP1 silencing altered the morphological and echocardiographic analysis in the ISO‐infused mice *in vivo*. Treatment with XBP1 silencing had no significant difference on heart size and myocyte size (Fig. 2B–C); however, XBP1‐silenced mice exacerbated cardiac hypertrophy after ISO infusion as evidenced by ANP mRNA expression and interstitial fibrosis (Fig. 2D–E). Meanwhile, ISO infusion also impaired both contractile functions and ventricular dilatation in control group mice. XBP1‐silenced animals developed significantly greater contractile impairment than the control group, despite reduced cardiac wall thickness (Fig. 2F–G). Taken together, these results indicate that an increase in myocardial XBP1 expression is protective against the detrimental consequences of ISO infusion.

### Myocardial capillary density and cardiac expression of pro‐angiogenic factor VEGF‐A is decreased in ISO‐treated mice after AAV9‐XBP1 shRNA treatment

**Figure 3:**
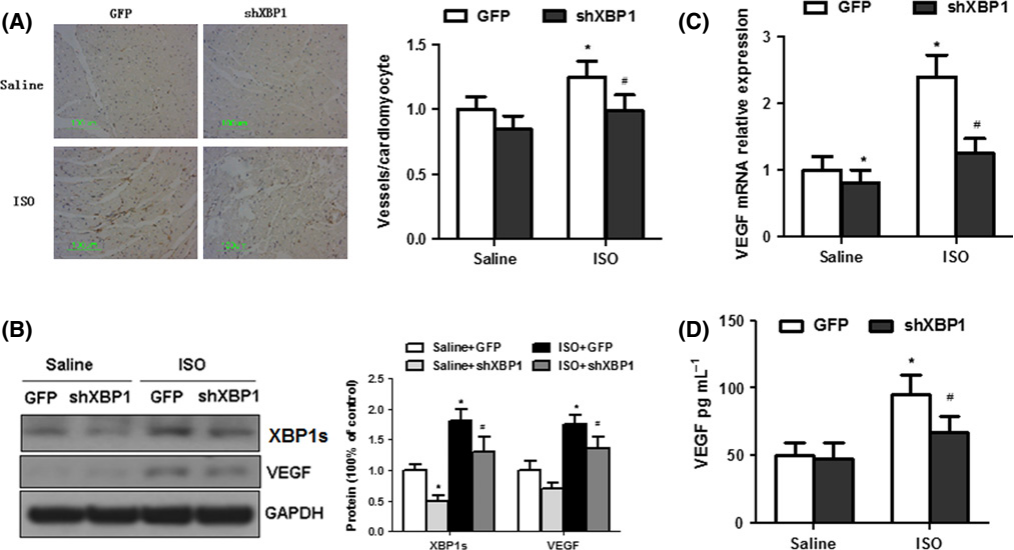
Myocardial capillary density and cardiac expression of pro‐angiogenic factor VEGF‐A is decreased in ISO‐treated mice after AAV9‐XBP1 shRNA treatment. (A) The number of microvessels per cardiomyocyte in ISO‐treated mice after AAV9‐XBP1 shRNA treatment. (B) Western blot analysis of VEGF and XBP1 expression in ISO‐treated hearts, with or without infection of AAV‐shRNA XBP1. *n* = 4. (C) Real‐time PCR analysis of VEGF mRNA expression in ISO‐treated hearts, with or without infection of AAV‐shRNA XBP1. *n* = 8. (D) ELISA of VEGF in blood sample of ISO‐infused mouse hearts after AAV‐shRNA XBP1 treatment. Error bars indicate SEM. *n* = 8 per group. The blot is representative of at least four blots from four independent experiments; *P < 0.05 compared with GFP or #*P* < 0.05 compared with ISO+GFP. *n* = 4.

Angiogenesis is essential in maintenance of cardiac function in hypertrophic hearts (Sano *et al*., 2007). Immunohistochemical analysis showed that the numbers of CD31‐positive cells were significantly lowered in XBP1‐silenced hearts (Fig. 3A), indicating that myocardial capillary density was impaired after XBP1 inhibition. Previous studies show that VEGF is an essential angiogenic factor to promote cardiac angiogenesis and maintain cardiac functions in hypertrophic hearts (Tomanek, 1990; Giordano *et al*., 2001; Sano *et al*., 2007). To address the potential role of XBP1 in VEGF‐A expression, the levels of VEGF‐A in ISO‐infused mice with or without AAV9‐XBP1 shRNA treatment were determined. Concomitantly with dramatic expression of XBP1, protein and mRNA expression of VEGF‐A was markedly increased after ISO infusion, but was ablated in XBP1‐silenced mice (Fig. 3B–C). Furthermore, secreted VEGF‐A in serum have same trend after XBP1 silencing treatment (Fig. 3D). These results suggest that XBP1 induction during stress is required to protect against ISO‐induced cardiac dysfunction by controlling the compensatory increase in cardiac VEGF‐A expression and angiogenesis.

### ER stress increases both XBP1 and VEGF‐A expression in cardiomyocytes

**Figure 4:**
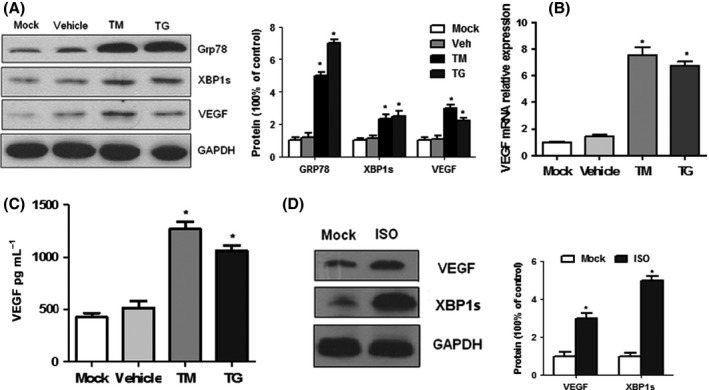
UPR increased VEGF‐A expression in H9C2 (2‐1) cells. (A) Western blots of XBP1s and VEGF in untreated and TM/TG‐treated H9C2 (2‐1) cells. (B) Real‐time PCR analysis of VEGF in H9C2 (2‐1) cells. (C) VEGF levels in culture supernatant from untreated and treated cells by ELISA. (D) Western blots of XBP1s and VEGF in untreated and ISO‐treated H9C2 (2–1) cells. Values are means ± SEM. The blot is representative of at least four blots from four independent experiments; *P < 0.05 compared with control. *n* = 4.

To investigate the possible role of UPR‐induced XBP1 in inducting pro‐angiogenic factor VEGF *in vitro*, we treated rat embryonic heart‐derived myogenic H9c2 (2‐1) cells with two pharmacological UPR inducers, thapsigargin (TG, a Ca2+ ATPase inhibitor that promotes ER stress by depletion of lumenal calcium stores) and tunicamycin (TM, which blocks protein glycosylation) for 24 h. Incubation of H9c2 cells with TM or TG markedly increased expression levels, as well as mRNA levels, of Grp78, spliced XBP1, and of VEGF‐A in cultured cardiac myocytes (Fig. 4A–C). We next determined the effects of ISO on XBP1 and VEGFA in cultured cardiomyocytes. As shown in Fig. 4D, ISO‐induced UPR attained similar results in H9c2 (2‐1) cells. Collectively, these results suggest that UPR synchronously induced upregulation of VEGF‐A and XBP1.

### UPR‐induced VEGF‐A upregulation depends on XBP1 *in vitro*

**Figure 5:**
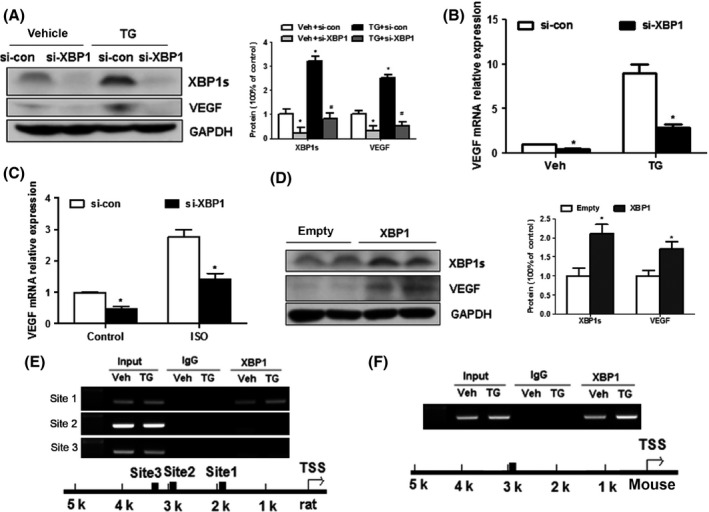
UPR‐induced VEGF‐A upregulation depends on XBP1 in H9C2 (2‐1) cells. (A) Western blots of XBP1s and VEGF in untreated and TG‐treated H9C2 (2‐1) cells after XBP1 siRNA transfection. (B), XBP1 siRNA (100 nM) knockdown of VEGF mRNA, with or without TG treatment in H9C2 (2‐1) cells, confirmed by real‐time PCR analysis. (C) XBP1 siRNA (100 nM) knockdown of VEGF mRNA, with or without ISO treatment in H9C2 (2‐1) cells, confirmed by real‐time PCR analysis. (D) XBP1s reintroduction by pCMV6‐XL5‐XBP1s vector in H9C2 (2‐1) cells increased VEGF and XBP1s expression. (E) ChIP assays of extracts from H9C2 cells treated with TG using anti‐XBP1s antibody (site 1: 1930bp). (F) ChIP assays of XBP1s on mouse VEGF promoter in untreated and TG‐treated MEFs (binding site: 2876 bp). Values are means ± SEM. The blot is representative of at least four blots from four independent experiments; *P < 0.05 compared with control. *n* = 4.

To further test whether XBP1 is involved in ER stress‐mediated VEGF‐A induction, siRNA targeting rat XBP1 (siRNA‐XBP1) was transfected into H9c2 (2‐1) cells with or without TG treatment. The results showed that the siRNA targeting XBP1 also synchronously reduced the mRNA and protein levels of spliced XBP1 and VEGF‐A (Fig. 5A–B). This induction of XBP1 and VEGF of ISO treatment is similar to that of TG treatment. Conversely, we next determined if ectopic expression of XBP1 altered VEGF expression in H9c2 (2‐1) cells. Compared to those transfected with control vectors, overexpression of XBP1s by transient transfection of pCMV6‐XL5‐XBP1s plasmid markedly increased VEGF expression in cultured H9c2 (2‐1) cells (Fig. 5D). Recently, XBP1 was reported to bind to the promoter regions of VEGF‐A in cancer cells (Roybal *et al*., 2005; Pereira *et al*., 2010). To confirm whether XBP1 is a transcription factor of VEGF in cardiomyocytes, we performed the Chromatin immunoprecipitation (ChIP) analyses and found that one putative XBP1s‐binding site (~1.9 kp) in VEGFA gene promoter was positively amplified after immunoprecipitation with primary antibody to XBP1, and TG increased this binding in H9C2 cells (Fig. 5E). Taken together, these data indicate that VEGF is a direct transcription target of XBP1 in cardiomyocytes. Furthermore, to characterize the relevance of the above observations with mouse cell line, we performed the ChIP assay and detected similar effects of XBP1 on VEGF regulation in mouse embryonic fibroblast (MEF) (Fig. 5F).

### Upregulated VEGF‐A is correlated with XBP1s in human hypertrophic and failing heart

**Figure 6:**
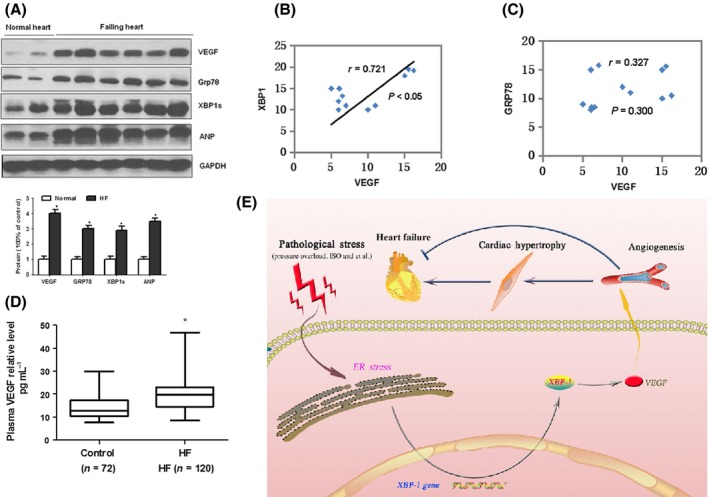
Upregulated VEGF in human heart failure heart samples is correlated with the increases of XBP1. (A) Western blot analysis of cardiac XBP1s, Grp78, VEGF, and ANP expression in human failing heart samples. (B–C) Increased VEGF expression in human heart failure heart samples is correlated with the increases of ER stress markers XBP1s but not Grp78. (D) ELISA of VEGF in blood sample of heart failure patients. The blot is representative of at least four blots from four independent experiments; *P < 0.05 compared with control. (E) Proposed mechanism underlying the transition from cardiac hypertrophy to heart failure. Deregulation of XBP1 suppresses VEGF‐mediated angiogenesis, which may contribute to the progression from adaptive hypertrophy to heart failure.

Finally, to characterize the relevance of the above observations of animal model and cardiomyocytes, we further measured the expression of UPR transcription factor XBP1, the ER chaperone GRP78, and the pro‐angiogenic factor VEGF in human myocardial samples by Western blot analysis. And the results showed that ER stress marker GRP78 and XBP1 were significantly increased in all failing human hearts compared to normal hearts, as well as ANP, a marker associated with cardiac hypertrophy and heart failure (Fig. 6A). As expected, the level of VEGF also was significantly elevated in failing hearts when compared to donor normal hearts (Table 1). The increase of myocardial VEGF was significantly correlated with the increase of XBP1 but not Grp78 (Fig. 6B–C). Then, we collected plasma samples from 120 patients with heart failure and 72 healthy adults to investigate circulating VEGF levels and found that the circulating expression levels of VEGF measured by ELISA were significantly increased in HF patients when compared to normal control (Fig. 6D).

**Table 1:** Characteristics for the study population

Characteristics	HF Cases(*n* = 120)	Normal(*n* = 72)
Age (years)	65 ± 15	60 ± 10
Male/female (*n*/*n*)	83/37	40/32
Current smoking, *n* (%)	36 (30%)	18 (25%)
Hypertension, *n* (%)	68 (56.67%)	15 (20.8%)
Fasting glucose (mm)	6.808 ± 1.36	5.2 ± 0.68
SBP (mmHg)	137.7 ± 24	125.2 ± 18
DBP (mmHg)	76.6 ± 12	65.5 ± 7
Ejection fraction (%)	46.2 ± 9.2	62 ± 7.8

SBP, systolic blood pressure; DBP, diastolic blood pressure.

Mean ± SD.

Taken together, these results raise the intriguing possibility that increased expression of XBP1 actually causes accumulation of VEGF protein and myocardial angiogenesis and contribute to the progression of cardiac hypertrophy (Fig. 6E).

## Discussion

In the present study, we have demonstrated that cardiac expression of ER stress transcription factor XBP1 was upregulated in pressure‐overload‐ and ISO‐induced cardiac hypertrophic mice. In addition, we found that increased XBP1 promotes VEGF‐A expression while silencing XBP1 inhibits VEGF‐A expression in cardiomyocytes. Furthermore, genetic inhibition of XBP1 inhibits cardiac VEGF expression and angiogenesis and exacerbates ISO‐induced cardiac dysfunction *in vivo*. Overall, our results indicate that an abnormal increase of XBP1 in the early stage of hypertrophic and failing heart is important in regulation of both the pro‐angiogenic factor VEGF and cardiac angiogenesis *in vivo*.

One of the most important findings of the present study is that XBP1 is essential for VEGF‐A upregulation and cardiac angiogenesis. The evidence can be summarized as follows: First, the levels of VEGF‐A and angiogenesis were correlated with the levels of XBP1s and ER stress in ISO‐infused hypertrophic hearts and failing human hearts. Second, inhibited expression of XBP1s decreased VEGF‐A expression and the number of CD31‐positive cell in hypertrophic and failing heart, demonstrating the pro‐angiogenesis property of XBP1s *in vivo*. Third, ISO increases both XBP1 and VEGF‐A expression in cardiomyocytes and silencing of XBP1 blocks ER stress‐induced VEGF expression in cardiomyocytes and reduces endothelial cell proliferation, migration, tube formation, and angiogenesis (Duan *et al*., 2015b), supporting that XBP1 silencing decreased cardiac capillary‐like network formation. Certainly, future research using AAV‐mediated restoration of XBP1 in maladaptive cardiac hypertrophy is required to provide further evidence of the biological role of XBP1 in the development of heart failure. Recent research further shows that XBP1 is a coregulator of HIF1α and controls the HIF1α transcriptional program in breast cancer (Chen *et al*., 2014). Interestingly, our recent study further found that cardiac expression of VEGF‐A, is correlated with XBP1s, was increased in the early adaptive phase, but decreased in the maladaptive phase in rat pressure‐overload‐ and isoproterenol‐induced hypertrophic and failing heart (Duan *et al*., 2015a). These researches support our finding that XBP1 is essential for VEGF induction in heart tissue.

On the other hand, a recent study reported that VEGF activates XBP1 mRNA splicing in endothelial cells via the AKT/GSK/β‐catenin/E2F2 signal pathway to promote endothelial cell proliferation and angiogenesis (Zeng *et al*., 2013), which supports and explains our findings on how secreted VEGF from cardiomyocyte regulates endothelial cell angiogenesis. There may be a XBP1‐VEGF‐XBP1 regulatory circuit from cardiomyocyte to endothelial cell in the process of cardiac angiogenesis. Importantly, Xbp1s was shown to be sufficient and necessary to protect heart from ischemia/reperfusion (I/R) injury *in vivo* (Wang *et al*., 2014). Our data are consistent with other investigations and also demonstrate that XBP1 is essential for tissue angiogenesis under physiological or pathological conditions (Romero‐Ramirez *et al*., 2009; Zeng *et al*., 2009, 2013; Ghosh *et al*., 2010; Ruan *et al*., 2013; Miyagi *et al*., 2013; Wang *et al*., 2014) and that XBP1 is an important regulator of vascular function and cardiac angiogenesis. In recent years, previous studies have shown that as a key stress‐inducible transcription factor in mammalian cells, XBP1 splicing plays an important role in the regulation of cell survival (Thuerauf *et al*., 2006), inflammation (Martinon *et al*., 2010), insulin sensitivity (Ozcan *et al*., 2004), glucose homeostasis (Ozcan *et al*., 2004; Zhou *et al*., 2011), lipogenesis (Lee *et al*., 2008; So *et al*., 2012), and autophagy (Margariti *et al*., 2013). Here, uncovering the exact molecular mechanisms of XBP1s‐induced cardioprotection will require further investigation.

Vascular endothelial growth factor (VEGF) is an essential angiogenic factor to promote angiogenesis and neovascularization and regulate all types of vascular growth and has thus received much attention regarding their potential use for therapeutic vascular growth in cardiovascular diseases (Ng *et al*., 2006). Previous studies have shown that VEGF‐B gene transfer resulted in prevention of the angiotensin II‐induced diastolic dysfunction associated with induction of the Akt pathway (Serpi *et al*., 2011), while VEGF blockade promotes the transition from compensatory cardiac hypertrophy to failure in response to pressure overload (Izumiya *et al*., 2006). Our data also show that the circulating expression levels of VEGF were significantly increased in heart failure patients, suggesting the plasma concentration of VEGF can be a potential indicator for heart failure. Certainly, further research is warranted to establish whether plasma levels of VEGF were related to different cardiac function. Interestingly, it is confused about that there is an unmatched phenotype between cardiac hypertrophy and cardiac function in mice with VEGF‐assisted treatment. Possible explanation was that the upregulation of VEGF‐A expression increases the capillary/myocyte ratio, but still leads to a net reduction in capillary density (capillaries mm^−2^), because the increase in capillarization (capillaries/myocyte) cannot keep match with myocyte growth (myocyte cross‐sectional area). When inhibition of cardiac angiogenesis further reduces capillary/myocyte ratio and leads to a greater reduction in coronary capillary density, contractile function, increased LVED dimension, ANP expression, and interstitial fibrosis contributed to a rapid transition to heart failure (Izumiya *et al*., 2006).

Previous studies have shown that XBP1s and VEGF were involved, respectively, in diverse cellular functions and processes (Ng *et al*., 2006; Glimcher, 2010). Now, our study linked these two different pathways and offered a new insight to investigate the physiologic and pathophysiologic significance of the XBP1/VEGF axis in multiple human diseases. Base our present study, XBP1s/VEGF‐A was correlated with cardiac angiogenesis in the progression from adaptive hypertrophy to heart failure. In the future, we will continue to check the contribution of this pathway in the aging heart disease and myocardial infarction. In additional, both XBP1s and VEGF‐A can regulate the development of retinal vasculature (Zeng *et al*., 2013), may offer a potential therapeutic target in the treatment of pathological angiogenesis for retinal/choroidal diseases. Certainly, the XBP1s/VEGF‐A pathway also may have important implications in developing antiangiogenic tumor treatment strategies in cancer diseases (Koong *et al*., 2006). Importantly, as a novel regulator of metabolic homeostasis (Lee *et al*., 2008; Zhou *et al*., 2011; Wu *et al*., 2014), the proposed XBP1 regulation of VEGF also broadens our knowledge on the pathologies of metabolic syndrome and sheds light on the intervention of diabetes mellitus and obesity‐related metabolic diseases.

In summary, our study has for the first time established that XBP1 is an important pro‐angiogenic factor, required to maintain normal cardiac function in the early stage of hypertrophy which results in the transition of hypertrophic hearts to heart failure. Thus, modulation of XBP1 might be a valid therapeutic target to prevent or delay heart failure.

## Experimental procedures

### Materials and reagents

Antibodies against XBP1, Grp78, VEGF, GAPDH, and β‐actin were purchased from Santa Cruz Biotechnology Inc. (Santa Cruz, CA, USA); antibodies against CD31 were purchased from Abcam Inc. (Cambridge, MA, USA). Nonspecific negative control oligonucleotides and specific siRNA against human or rat/mice XBP1 were obtained from RiboBio (Guangzhou, China). All other chemicals and reagents were purchased from Sigma‐Aldrich China Inc. (Shanghai, China), unless otherwise specified.

### Cell lines

The embryonic rat heart–derived myogenic cell line H9c2 (2‐1) and mouse embryonic fibroblast (MEF) were obtained from the American Type Culture Collection (ATCC, Manassas, VA, USA). The H9c2 (2‐1) cells and MEFs were grown in Gibco DMEM medium supplemented with 10% fetal bovine serum (Gibco, Invitrogen, Carlsbad, CA, USA), streptomycin 100 μg mL^−1^, and penicillin 100 U mL^−1^ (all obtained from Sigma, St Louis, MO, USA). All cells were grown at 37 °C in an atmosphere of 5% CO_2_.

### Animal models

Eight‐week‐old male C57BL/6 mice were subjected to thoracic aorta constriction (TAC) or sham operation as described previously (Okada *et al*., 2004). Pressure‐overload‐induced cardiac hypertrophy and heart failure was created by TAC for 2 weeks. C57BL/6 mice were continuously infused with isoproterenol hydrochloride (15 mg kg^−1^ day^−1^; Sigma‐Aldrich China Inc.) over a period of 14 days with implanted minipumps. Mini‐osmotic pumps (Alzet model 1007D and 1002; DURECT Corp., Cupertino, CA, USA) were implanted as described previously (Nienaber *et al*., 2003). Mice were anesthetized with pentobarbital sodium at a dose of 80 mg kg^−1^ body weight intraperitoneally. Animals were euthanized via an anesthetic overdose (200 mg kg^−1^ of pentobarbital sodium delivered by intraperitoneal injection). All animal studies were approved by the Animal Research Committee of Tongji College and were carried out according to the guidelines of the National Institutes of Health (NIH).

### Echocardiography

Echocardiography was performed on mice with a VisualSonics Vevo 2100 System echocardiograph (VisualSonics, Toronto, CA, USA) using a 35‐MHz probe as described previously (Lin *et al*., 2009; Wang *et al*., 2011).

### Heart tissue samples

Human heart tissue samples were studied according to the protocol approved by the Clinical Research Committees of Tongji Medical College and the guidelines of the National Institutes of Health (NIH). The investigation also conforms to the principles outlined in the Declaration of Helsinki. The subjects recruited to the study provided informed consent. We collected heart samples from six recipients of heart transplantation who suffered end‐stage heart failure. The normal hearts were from victims of traffic accidents. Tissue samples were frozen at −80 °C until used for extraction of protein.

### Vector construction

Recombinant adeno‐associated virus vectors rAAV9‐GFP and rAAV9‐shXBP1 (rAAV9 generating shRNA to silence XBP1) were produced by a 2‐plasmid protocol described previously (Suckau *et al*., 2009) and then prepared and purified according to a published protocol (Inagaki *et al*., 2006). The siRNA sequences for mice XBP1 were: 5′‐CAC CCU GAA UUC AUU GUC U‐3′. The plasmid constructs were verified by sequencing.

### Gene delivery study *in vivo*

In C57BL/6 mice study, rAAV9 was used as carrier. C57BL/6 mice were injected with rAAV‐GFP or rAAV‐XBP1 shRNA (*n* = 8 per group) through a sublingual vein injection 2 weeks before ISO infusion as described previously (Masson *et al*., 1998; Feng & Li, 2010). Two weeks after ISO infusion, mice were subjected to echocardiography examination, followed by morphological and immunohistological tests.

### Immunohistochemical analysis

For immunohistochemical examination of CD31, paraffin‐embedded rat heart tissues were cut into 5‐μm sections, and assays were performed as previously described (Jiang *et al*., 2005). Antibodies for CD31 were used as the primary antibody.

### ChIP assay for XBP1 and VEGFA gene promoter binding

ChIP assays were performed according to the manufacturer's protocol using a ChIP kit (17‐295; Millipore, Temecula, CA 92590). The primers used for ChIP in H9C2 cells were described previously (Pereira *et al*., 2010). The primers used for ChIP in MEFs were as follows: 5′‐GCG TCA CTG CTC TGG CTC CCT GT‐3′ and the reverse primer 5′‐CGG GCC TAC AGA AGA GGG ACT CAG TA‐3′.

### Cell transfection

H9c2 cells were seeded in 6‐well plates at 1.5 × 10^5^ cells/well, 24 h before transfection. Cells were transfected with siRNA after seeding using Lipofectamine 2000 (Invitrogen) and transfected with plasmid using X‐tremeGENE HP DNA Transfection Reagent (Roche, Indianapolis, IN, USA), according to the manufacturer's recommendations. Transfections contained 50 or 100 nm small interfering RNAs (siRNAs) against XBP1 and siRNA control (RiboBio) in a final volume of 2 mL. The siRNA sequences for rat XBP1 were as follows: 5′‐GAG AAA GCG CUG CGG AGG A‐3′ (Sawada *et al*., 2010). Cells were collected 48 h after transfection for protein and RNA extraction.

### VEGF secretion assay

After cells were exposed to 24 h of TM or TG, the VEGF‐A protein concentrations in the culture medium were measured by ELISA using a human or rat VEGF‐A ELISA kit (R&D Systems, Abingdon, UK) according to the manufacturer's protocol.

### RNA extraction and real‐time quantitative PCR

Total RNA was extracted using TRIzol, according to the manufacture's protocol. Reverse transcription reactions were performed starting from equal amounts of total RNA/sample (1 μg) using EasyScript First‐Strand cDNA Synthesis SuperMix (TransGen Biotech, Beijing, China). Rat VEGF mRNA levels were determined using the forward primer 5′‐CAG CTA TTG CCG TCC AAT TGA‐3′ and the reverse primer 5′‐CCA GGG CTT CAT CAT TGC A‐3′ (Romero‐Ramirez *et al*., 2009); mice VEGF mRNA levels were determined using the forward primer 5′‐TGT ACC TCC ACC ATG CCA AGT‐3′ and the reverse primer 5′‐TGG AAG ATG TCC ACC AGG GT‐3′; β‐actin or GAPDH was used as an internal reference. Results of the real‐time quantitative PCR were analyzed and expressed as relative mRNA levels of the cycle threshold (CT) value, which was then converted to fold change.

### Western blot analysis

Proteins from cell lysates (20 μg) were separated by 10% SDS–polyacrylamide gel electrophoresis and transferred to a polyvinylidene difluoride membrane. After blocking in 5% nonfat milk, protein blots were incubated with a specific antibody followed by incubation with a peroxidase‐conjugated secondary antibody in blocking buffer. The bands were visualized using the enhanced chemiluminescence method, according to manufacturer's instructions (Beyotime Institute of Biotechnology, Haimen, China).

### Semiquantitative reverse transcription–PCR

To detect rat unspliced and spliced XBP1, the primers were 5′‐AAA CAG AGT AGC AGC ACA GAC TGC‐3′ and 5′‐TCC TTC TGG GTA GAC CTC TGG GAG‐3′ (Lai *et al*., 2008). After 35 cycles, PCR products for rat unspliced (XBP1u, 480 bp) and spliced (XBP1s, 454 bp) XBP1 fragments were separated by 2% agarose gels stained with GoldView. The relative intensity of XBP1 compared with β‐actin was calculated for each sample by densitometry.

### Statistical analysis

The data are expressed as mean values ± SD. Differences between groups were evaluated for significance using Student's *t*‐test of unpaired data or one‐way analysis of variance (anova) and Bonferroni post‐test. *P* < 0.05 was considered significant.

## Funding info

This study was supported by grants from the National Natural Science Foundation of China (30770882, 81470519, 31400998, 91439203, and 31130031) and National Basic Research Program of China (973 Program, No. 2007CB512004). The funders had no role in study design, data collection and analysis, decision to publish, or preparation of the manuscript.

## Author contributions

The author(s) have made the following declarations about their contributions: Quanlu Duan and Dao Wen Wang conceived and designed the experiments. Quanlu Duan, Lei Yang, Ben Ma, and Wei Gong performed the experiments. Quanlu Duan, Chen Chen, and Li Ni analyzed the data. Quanlu Duan, Peihua Wang, and Zhejun Cai contributed reagents/materials/analysis tools. Quanlu Duan, Dao Wen Wang, and Ming‐Hui Zou wrote the manuscript.

## Conflict of interest

The authors have declared that no competing interests exist.
